# Assessment of the Distribution, Sources and Potential Ecological Risk of Heavy Metals in the Dry Surface Sediment of Aibi Lake in Northwest China

**DOI:** 10.1371/journal.pone.0120001

**Published:** 2015-03-17

**Authors:** Jilili Abuduwaili, Zhao yong Zhang, Feng qing Jiang

**Affiliations:** 1 State Key Laboratory of Desert and Oasis Ecology, Xinjiang Institute of Ecology and Geography, Chinese Academy of Sciences, Urumqi, China; 2 University of the Chinese Academy of Sciences, Beijing, China; Institute of Tibetan Plateau Research, CHINA

## Abstract

The distribution, sources and potential ecological risk of heavy metals in the sediment of lakes in eastern China and other areas of the world that have undergone rapid economic development have been widely researched by scholars. However, this is not true for heavy metals in the sediment of rump lakes in the arid regions of China and world-wide. Because of this, we chose Aibi Lake to serve as a typical rump lake in an oasis in an arid area in northwest China for our study. Sediment samples were collected from the lake and then the quantities of the heavy metals Pb, Ni, Cd, Cu, Zn, Hg and Cr were measured. Then using a variety of statistical methods, we analyzed the distribution, sources, pollution status and the potential ecological risk of these metals. The results show that: (1) The amounts of the seven heavy metals all fell within the Second Soil National Standard, but the average and maximum values were all higher than the background values of Xinjiang in northwest China. (2) Multivariate statistical analysis determined that the Cd, Pb, Hg and Zn in the sediment were mainly derived from man-sources, and Cu, Ni, and Cr were mainly from the natural geological background. (3) Enrichment factor analysis and the geo-accumulation index evaluation method show that Cd, Hg and Pb in the surface sediment of the Aibi Lake were at low and partial pollution levels, while Zn, Cr, Ni and Cu were at no and low pollution levels. (4) Calculation of the potential ecological hazards index found that, among the seven tested heavy metals, Cd, Hg and Pb were the main potential ecological risk factors, and the contribution of each was 42.6%, 28.6%, and 24.0%, respectively. Cd is the main potential ecological risk factor, followed by Hg and Pb. This work revealed that recent economic development of the Aibi Lake Basin has negatively influenced the accumulation of heavy metals in the sediments of the lake, and, therefore, we should pay increasing attention to this problem and take effective measures to protect the ecology of the Aibi Lake Basin. This work can provide a scientific basis for an early warning of heavy metal pollution and for protection of the environment. Furthermore, it can serve as a reference when creating policies for the economic development in Aibi Lake Basin and environmental protection of rump lakes in arid regions of northwest China and other areas of the world.

## Introduction

Heavy metals are concerning pollutants because they are not biodegradable and, once they reach a certain concentration, are quite harmful to the environment [1,2,3]. Lakes are one place where heavy metals are a concern as the accumulation of heavy metals in the sediment of lakes results in pollution that can accumulate in organisms, where it can become toxic. Thus, this makes heavy metals a potential risk to human health through the food chain [4,5]. In addition, heavy metals pose a potential ecological risk. Due to hydrodynamic condition changes, biological disturbances, physical and chemical conditions and a series of complicated processes, heavy metals will release from sediments and water to produce “Secondary Pollution”[6,7].

Human activity plays an important role in heavy metal pollution in lake sediments. Along these lines, different types and intensities of activity in the lake basin may lead to significantly different heavy metal pollution statuses for the lake sediment [8,9,10]. Where the primary activity is agricultural in the lake basin and the amount of human activity is less, then the degree of heavy metal pollution in the lake sediment is relatively low. When lakes are near urban and industrial areas, the diversity and strong intensity of human activity results in heavy metal pollution that is generally more serious[11,12]. With the recent rapid economic development in China, many lakes have become polluted with heavy metals. This pollution is to a higher degree in lakes in the eastern parts of the country. These areas have gained increasing attention from scholars. Qiao et al. (2005) [8] assessed the potential ecological risk posed by heavy metals in the sediments of six lakes in the Wuhan region of southeast China. Chen and Li, (2007) [13] measured and analyzed the total amount and morphological fraction of ten heavy metals in the sediments of the Chao hu in eastern China and discussed the activity of different forms of heavy metals. They found the bioavailability of Cd was much higher than other heavy metals in the sediment and was a result of agricultural pollution. Zeng and Wu, (2007) [14] using geochemical and statistical methods to distinguish between the anthropogenic sources of heavy metals in the sediment of Fuxian Lake in the Yunnan province of southwest China, found that man-made pollution has become a serious environmental problem in this lake.

In the lakes of the arid regions in northwest China, research on the amounts and proportions of heavy metals in lake sediments is lacking [15–17]. In Xinjiang in the typical arid regions of northwest China, Zhang et al. (2009) [18]analyzed the amount of heavy metals, the pollution levels and the changing trends in sediments of Bosten Lake in south Xinjiang of northwest China. They found a low level of Cu, Ni, Co and Ni pollution in the southern part of the lake, which mainly derived from rock weathering and other geochemical processes. However, there was also a moderate amount of Pb and Cd pollution that mainly came from the use of phosphate fertilizers in agriculture. Since the 1960s, the amounts of Pb and Cd in the sediment of the lake has significantly increased. Zheng and Luo, (2011) [19] recently researched the changes in metals in the water of Barkol Lake and found that the amount of Cu in the lake sediment was significantly higher in 2008 than in 2001 that are significantly higher than the First Water Quality Standard of China (GB 3838–2002) [20], indicating economic activities over those years in this area had negatively influenced heavy metal accumulation in the lake sediment.

Aibi Lake (43°38′-45° 52′N; 79° 53′-85°02′E) is located in the western part of the Junggar basin in Xinjiang in northwest China, and is a typical rump lake in an arid region [21,22]. It also forms a closed basin called Aibi Lake Basin. The basin is considered an important base for grain, cotton, animal husbandry, oil and chemical industries. In Xinjiang in northwest China, it is also an important open channel to the west, known as the “Euro-Asian Continental Bridge” that runs along the Aibi Lake across the lake basin from the north to the southwest [23,24]. Since the 1990s, both the implementation of the “Western Development Policy of China” and the developmental policy by the Xinjiang Uygur Autonomous Region in northwest China has led to prodigious economic development in the Aibi Lake Basin. In this area, fertilizers and pesticides imprudently used in agricultural processing and large quantities of emissions by the townships have led to pollution of the main feeders, the Jing and Bortala Rivers. This has significantly and negatively influenced the water quality of the main water area of Aibi Lake. This pollution will eventually seep into the sediment, and become a threat against the aquatic organisms and the ecological environment [25].

Based on this, we chose a typical rump lake in an arid region of northwest China, Aibi Lake, for our study. Using ArcGIS 10.0 software and combining the grid sampling method with 3S technology, we get a total of 43 sediment samples, then we tested the total quantity and chemical fraction of the seven heavy metals of Hg, Pb, Zn, Cu, Cr, Cd and Ni in the sediments of the lake. Classical statistics, multivariate statistical analyses and enrichment factor (*EF*) methods were then used to study the occurrence and sources of these heavy metals. To determine the pollution status of and potential ecological risk posed by each heavy metal, the geological accumulation index method (*I*_*geo*_) and potential ecological risk evaluation method (*RI*) by Hakanson (1980) [26] were used, respectively. A comparison was then performed between the amount of each of the heavy metals in the sediment of the test lake with other lakes in China and other regions of the world. By analyzing the spatial distribution characteristics of *EF*_*S*_, *I*_*geo*_, and the land use types, rivers, townships, traffic arteries and meteorological factors of the Aibi Lake Basin, we determined the primary factors influencing the distribution of heavy metals in this area. This work is a scientific basis for controlling pollution of Aibi Lake, and can serve as a reference for protecting the Aibi Lake Basin as well as other basins in the arid regions of northwest China.

## Materials and Methods

### Study area

**Fig 1:**
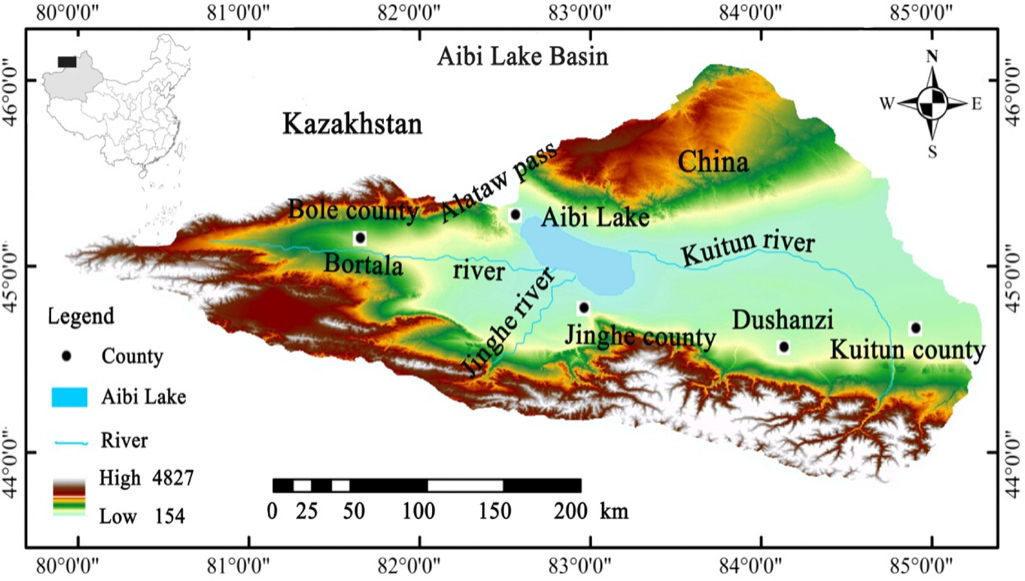
Map showing Aibi Lake Basin.

The Aibi Lake Basin is a closed basin located in the inland area of the Junggar basin in Xinjiang in northwest China (43°38′-45°52′N, 79°53′-85°02′E) (Fig. 1). It has a total area of 50621 km^2^, where plains make up 25762 km^2^ and lakes 542 km^2^. The Aibi Lake is the largest lake in the basin as well as the largest salt lake in Xinjiang in northwest China with a water area of 520 km^2^. Due to the arid desert climate, there is little rainfall with the average annual rainfall of the basin around only 100–200 mm and a potential evaporation of up to 1500–2000 mm. The Bortala, Jing he and Kuitun Rivers are the main feeders into the lake that come from the west, south and east, respectively. Over the last 50 years, a combination of the population and large-scale exploit of water and soil of Aibi Lake has led to a dramatic reduction in the amount of water flowing into the lake from rivers [25]. In the Aibi Lake Basin, the flora is primarily that of Central Asia and Mongolia. There is a total of 385 plant species belonging to 53 families and 191 genera [27]. The soil types are mainly *Piedmont psephitic* and *Gypsum desert soil*, the vegetative cover is mainly *H*. *ammodendron desert* and *Ephedra desert* and plant growth is *Populus euphratica forest*, *Phragmites australis* and lowland meadow. Since the 1950s, the driving forces of industrial and agricultural development have dramatically decreased the amount of water flowing into the lake, resulting is a reduction in the lake water area from 1000 km^2^ to 500 km^2^. As the lake has dried up, the ecological environment of the lake basin has visibly deteriorated [28].

### Sediment sampling and analyses

The samples collection and test all permitted in our country and no specific permissions were required for these locations/activities, we confirm that the field studies did not involve endangered or protected species. First, we performed a basic analysis of the water depth and topography of the bottom of Aibi Lake. Then, we used ArcGIS 10.0 software to lay out a grid of sediment sampling points, resulting in 43 sediment samples. All samples were collected in the dry lakes bottom, during field sampling, we adjusted the sample sites based on the actual environment. The samples were then put into clean polyethylene plastic bags. All samples weighed >400g. During the sample processing, the collection site, date, and the sediment color of each sample were recorded.

The amount of each heavy metal in the samples was measured as follows: 0.5 g sample was placed into an Anton PVC digestion tank along with HF-HCl-HNO_3_^-^. The tank was sealed and the sample was incubated at 170°C for 30 min. An inductively coupled atomic spectrum emission spectrometer (ICAP 7500, Dionex Corporation, USA) was used to measure the Pb, Ni, Cd, Cu, Zn and Cr contained in the samples. An Atomic fluorescence spectrometer (Atomic Fluorescence Spectrometry, AFS) was used to measure Hg.

First, standard curves were obtained using separate solutions containing known concentrations of each heavy metal (GSS series, China) that had been diluted with deionized water. Next, these standard curves were used to optimize the machine as well as measure the heavy metals. Finally, in order to verify the accuracy of our heavy metal measurements, 10% of the samples were retested. The standard solutions of the heavy metals were used to compare our samples to national standards (GSS series, China). The recovery of each heavy metal in all the samples was 96.5%-101.9%. To prevent contamination during the testing process, all glassware was soaked in 5% HNO_3_ for 24 hours, rinsed and then dried. All reagents were of analytical grade and were used without further purification and all solutions were prepared with Milli-Q water. Blanks and duplicates were regularly employed during testing.

### Statistical analyses

#### Enrichment factor (EF) method

*EF* is an important indicator that quantitatively assesses the levels and sources of heavy metal pollution. *EF* is calculated as displayed below [29, 30]:

$$EF={\left(c_{i}/c_{ref}\right)}_{samples}/{\left(B_{i}/B_{ref}\right)}_{baseline}$$ (Eq. 1)

Where *EF* is the enrichment level of a certain heavy metal, *C*_*i*_ is the measured concentration of *i* heavy metals in the sediment, *C*_*ref*_ is the measured concentration of the reference element, *B*_*n*_ is the background value of the local region and *B*_*ref*_ is the background concentration of the reference element of the soil in the same region.

In the research, the background value of Cd, Cr, Cu, Hg, Ni, Pb and Zn of the local region (*B*_*n*_), were chose from the supra-crust of western Jounggar region, Xinjiang, because the Aibi lake was located in this regions of Jounggar basin, and the reference elements are much proper than the background values of Xinjiang, China, and eventually we get the background values of Cd, Cr, Cu, Hg, Ni, Pb and Zn of Aibi lake basin are 0.08, 26.49, 32.37, 9.27, 14.25, 11.19, and 110.6 mg/kg [31].

According to criteria published by Sutherland (2000) [32], when *EF*<2, there is no enrichment of these heavy metals and they mainly derive from the natural environment. When 2<*EF*<10, there is a moderate enrichment of the heavy metals and this pollution comes from man-made sources. When 10<*EF*, there is a significant enrichment of the heavy metals and the man-made pollutants are most likely their primary source [29, 30].

#### Geo-accumulation index (I_geo_)

*I*_*geo*_ is a quantitative indicator proposed by Muller (1969) [33] that estimates the heavy metal pollution status of sediment in water. This method directly reflects the degree of enrichment of heavy metals in sediment and the equation is displayed below.

$$I_{geo}=Log_{2}[C_{i}/1.5B_{i}]$$ (Eq. 2)

Where *C*_*i*_ is the measured concentration of the sediment from a water body, *B*_*i*_ is the geochemical background value of a particular heavy metal, and 1.5 is the coefficient of variation that may be caused by earth movement and rock formation. In this study, we chose the average values of global shale as the background reference values. The values of Cd, Cr, Cu, Hg, Ni, Pb and Zn were 0.3 mg.kg^-1^, 90 mg.kg^-1^, 45 mg.kg^-1^, 0.017 mg.kg^-1^, 26.7 mg.kg^-1^, 26.6 mg.kg^-1^ and 19.4 mg.kg^-1^, respectively [34].

Based on the published literature, the *I*_*geo*_ criteria of heavy metals in the sediment in this work are shown in Table 1 [35,36].

**Table 1:** Contamination levels and classification based on *I*_*geo*_ of heavy metals.

*I*_*geo*_	≤0	0–1	1–2	2–3	3–4	4–5	>5
Classification	0	1	2	3	4	5	6
Pollution level	Clean	Slight	Partially moderate	Moderate	Partially serious	Serious	Severe

#### Potential ecological risk index (RI)

The *RI* was proposed by Hakanson in 1980 [26] and is used to evaluate the potential ecological risk posed by heavy metals in the sediment of water bodies. This comprehensive method considers the four factors of concentration, types of pollutant, toxicity level and the sensitivity of the water body to metal contamination of the sediment. This method is now widely used by scholars around the world to evaluate the potential ecological risk of heavy metals in sediments [37,38,39,42,43]. The equation to calculate potential *RI* is displayed below.

$$RI=\overset{I}{\underset{M}{\sum^{\text{​}}}}E_{r}^{i}=\overset{I}{\underset{M}{\sum^{\text{​}}}}T_{r}^{i}\times C_{r}^{i}=\overset{I}{\underset{M}{\sum^{\text{​}}}}T_{r}^{i}\times \frac{{\text{c}}^{i}}{{\text{c}}^{i}{}_{n}}\;$$ (Eq. 3)

Where *RI* is the potential ecological risk index of heavy metals in sediment, *E*_*r*_^*i*^ is the potential ecological risk coefficient of a particular heavy metal, *C* ^*i*^_*r*_ is the pollution factor, *C* ^*i*^ is the amount of heavy metal, c^*i*^_*r*_ is the reference value of heavy metal *i*. To facilitate comparisons between the background of and the studies on Xinjiang in northwest China, we used the background values of Xinjiang as reference values in this work [40]. *T*_*r*_^*i*^ is the toxicity coefficient of specific single pollutants and can reflect the toxicity, pollution levels and the sensitivity of the environment to heavy metals. Based on related published research, the toxicity coefficients of Pb, Ni, Cd, Hg, Cu, Zn and Cr in this study were 5, 5, 5, 40, 5, 5 and 2, respectively [41].

## Results and Analysis

### Descriptive statistical analysis of heavy metals

The descriptive statistics calculated in this work are shown in Table 2. Specifically, the maximum average values of Cd, Cr, Cu, Hg, Ni, Pb, and Zn in the sediment of Aibi Lake were 0.72 (0.17), 262.81 (51.49), 95.84 (39.89), 1.94 (0.033), 58.18 (28.27), 175.81 (39.57) and 217.27 (114.59) mg.kg^-1^, respectively. This analysis resulted in average values for the seven heavy metals that were all within the Secondary National Standard of China [44] (when pH>7.5, the value of Cu is the agricultural standard). However, the maximum and average values of the heavy metals all exceeded the background levels of Xinjiang [40] and the maximum values of Cd, Hg and Cr exceeded the Secondary National Standard of China[44]. This indicates that, due to the agricultural and human activity in townships in the Aibi Lake Basin, there has been a significant trend of heavy metal accumulation in the sediment of Aibi Lake.

**Table 2:** Descriptive statistics of the heavy metals in surface sediment from Aibi Lake.

Elements	Range (mg.kg^-1^)	Mean (mg.kg^-1^)	SD (mg.kg^-1^)	CV (%)	O-L (%)	Kurtosis	Skewness	BV (mg.kg^-1^)	NSS (mg.kg-1)
Zn	88.27–217.27	114.59	31.79	72.87	22.51	22.5	19.4	68.8	300
Pb	16.29–175.81	39.57	5.18	85.84	28.28	25.4	22.4	19.4	350
Cu	32.29–95.84	39.89	3.54	28.65	15.27	19.6	15.4	26.7	100
Hg	0.01–1.94	0.033	2.27	95.49	24.41	43.8	37.3	0.017	1.0
Ni	11.81–58.18	28.27	4.28	24.81	18.27	22.2	14.6	26.6	60
Cd	0.01–0.72	0.17	0.46	101.51	15.64	33.4	29.6	0.12	0.6
Cr	45.41–262.81	51.49	2.95	32.42	14.19	20.2	14.7	49.3	250
Al	18.9–65.2	53.16	/	/	/	/	/	/	/

Annotation: SD is the standard deviation, CV is the variable coefficient, O-L is the exceed ratio of heavy metals for the background or the national soil standard, BV is the background values of the heavy metals, and NSS is the secondary national soil standard values.

According to the classification of variation of heavy metals by Lv et al. (2012) [45], the variations of Cu, Ni, Cr, and Zn were 28.65%, 24.81%,32.42% and 31.79%, respectively, put**t**ing them at medium variation. By contrast, the variation of Cd, Hg and Pb were 101.51%, 95.49%, and 85.84%, respectively, putting them all at high variation. Furthermore, the variable coefficients of Hg and Cd were obviously higher than the other elements tested, indicating their distribution among the samples was uneven and they were mainly influenced by artificial sources [46].

### Multivariable statistical analysis

#### Correlation analysis

**Fig 2:**
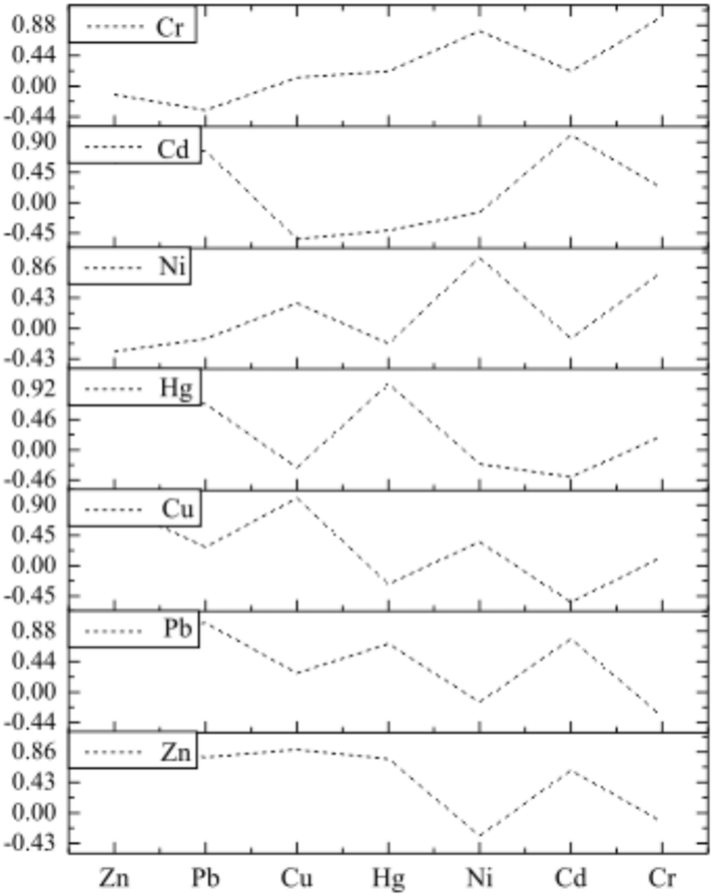
Correlation coefficients figure of heavy metals in surface sediment from Aibi Lake.

Correlation analysis results are shown in Fig. 2. Overall, the correlations between the seven heavy metals are complex with relatively high correlations between Hg, Zn and Pb. Among these, the correlations between Pb and Hg, and Pb and Cd were 0.698, and 0.771, respectively, and at a *P*<0.01 level, and, therefore, have a highly significant correlation. The correlations between Zn and Hg, Zn and Cu, and Zn and Cd were 0.759, 0.891, and 0.598, respectively, and significantly correlated at a *P*<0.05. The correlations between Ni and Cr was 0.797, suggesting they are highly significantly correlated at a *P*<0.01.

#### Principal component analysis

**Fig 3:**
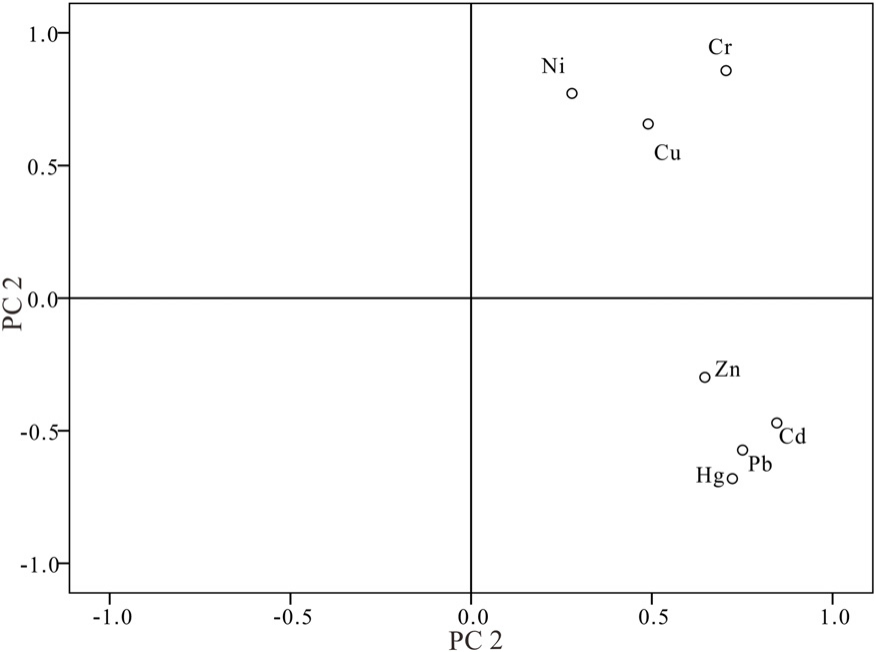
Principle components of heavy elements in surface sediment from Aibi Lake.

Principal component analysis (PCA) is an effective method to identify the sources of heavy metals in the environment [47,48]. In this work, the PCA results (Fig. 3) demonstrate that the seven heavy metals tested in the sediment from Aibi Lake can be categorized into two principal components (PCs). Overall, PC1 can explain 58.17% and PC2 29.38% of the total factors with a cumulative contribution rate of 90.09%, indicating this covers most of the sources of heavy metals in the sediment. There are large loads of Cd, Pb, Hg and Zn within PC1 and analysis revealed that the average value of each is higher than the background values of Xinjiang. By combining PCA with the soil sampling background, we can see that the samples with the highest amount of these four heavy metals were mainly distributed in the sampling sites located in the southern part of Aibi Lake. Based on this data and the related articles, we can conclude that the polluting emissions from townships and industries and the irresponsible use of fertilizers and pesticides in agriculture have contributed to the presence and eventual accumulation of Cd, Pb, Hg and Zn in water bodies, such as rivers, lakes, and bays [48,49]. There are high loads of Cu, Ni, and Cr within PC2 and strong correlations between these elements. The samples containing high amounts of Cu, Ni, and Cr were mainly from the sediment from the northern part of Aibi Lake, where the land types are mainly desert, bare land and alkaline soil, and are distant from traffic and townships. By combining related literature with our analysis, we can see the relatively high quantity of Cu, Ni, and Cr in the sediments of Aibi Lake mainly originate from the natural geological background of the lake bottom and Aibi Lake Basin_._

### Enrichment factor analysis (*EF*)

**Fig 4:**
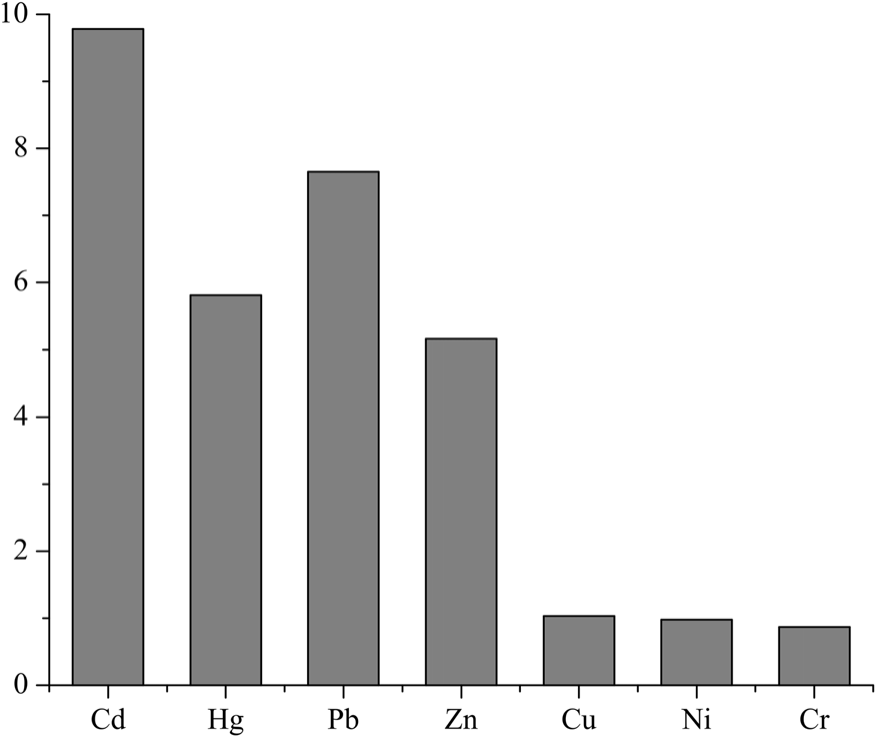
Enrichment factor (*EF*) of heavy metals in surface sediment from Aibi Lake.

*EF* was mostly used to analyze the enrichment of elements in the soil and the sediments (Equation 1) [29,30]. The average *EF* values for Cd, Pb, Hg and Zn in all the samples were 9.78, 7.65, 5.81 and 5.16, respectively. As they were all higher than 2, they suggest a relatively high level of pollution (Fig. 4) and their presence in the surface sediment of Aibi Lake was primarily from anthropogenic pollution. Taking into account all the sediment samples, the average *EF* values of Cu, Ni and Cr were 1.03, 0.98, and 0.87, respectively. Because these values are all lower than 2 and nearly 1, they suggest no pollution by Cu and Ni and low levels of Cr pollution.

### Distribution of *EF* of heavy metals in surface sediment from Aibi Lake

Based on the distribution of these polluting factors of heavy metals, we can see the sites with high *EF* values of Cd, Pb, Hg, and Zn were mainly distributed in the western, southern and southwestern parts of the lake, where there is the estuary of the Bortala and Jinghe Rivers in the west. Furthermore, this is also near the farmland, townships and high traffic areas in the south, resulting in exposure to human activity related to road traffic, urban lifestyles, and agriculture. The high *EF*_S_ values of Cr, Ni and Cu among the sediment samples were primarily distributed in the northern, central and northwestern areas of the lake. These are areas that have little human activity, are away from traffic arteries and townships, and include the land types of desert, bare land and saline-alkali soil. Analysis of the distribution of the enrichment of the seven heavy metals can be classified into two types consisting of Cd, Pb, Hg and Zn, and Cr, Ni and Cu. This result is consistent with the multivariate statistical analysis, indicating there are some relationships between them. In the text that follows, we will discuss possible reasons for the distribution of the heavy metals in the lake sediments as well as analyze the relationships between the distributions and the multivariate statistical analysis results.

### Geo-accumulation index evaluation (*I*_*geo*_) of heavy metals

Calculations of *I*_*geo*_ from all the sediment samples (Equation 2) resulted in *I*_*geo*_ values for Cd, Hg and Pb that were all higher than 0 and ranged from 0 to 3, thus putting them at low and partially moderate pollution levels. Among these metals, the pollution levels of Cd were the highest as the *I*_*geo*_ put Cd at low pollution levels in 72.1% of the samples, partially moderate pollution levels in 16.7% and moderate pollution levels in11.2%. For Hg, 76.1% of all samples fell into low pollution levels, 15.6% were at partially moderate pollution levels and 8.3% were at moderate pollution levels. The *I*_*geo*_ values of Zn, Cr, Ni and Cu in the sediments of Aibi Lake were 0.42, 0.29, 0.34 and 0.25, respectively. In general, the proportion of Zn, Cr, Ni and Cu suggests no pollution within the samples exceeding 60% of the samples. For low pollution status, the proportions of Zn, Cr, Ni and Cu were 37.5%, 14.7%, 11.8% and 18.3%, respectively, within the samples. However, Cd, Hg and Pb reached 72.1%, 76.1%, and 77.4%, respectively, a significantly high level, indicating most of these sediment samples were polluted. Based on the mean values of the pollution levels of the heavy metals, the order was Cd>Hg>Pb>Zn>Cu>Cr>Ni. Among these, Cd, Hg and Pb were significantly high at 27.9%, 23.9% and 22.6% of all samples as the *I*_*geo*_ values were above 1.

### Distribution of the pollution levels of heavy metals

The analysis showed Cd, Pb and Hg had high pollution indexes and were all located in the western and southern parts of the lake. The land types in this area include river estuary, farmland, townships and a traffic artery. The distribution of Cr, Ni, Zn and Cu suggested a high pollution index in the samples from the northern, central and northeastern parts of the lake, which were far away from the townships, farmland and traffic artery and lack an estuary. The land types were mainly desert and bare land. These analyses were consistent with the *EF* distribution of the heavy metals. Furthermore, the distributions of the seven heavy metals were classified into two types, which matched the multivariate statistical analysis results. The sources of these heavy metals were identified as from two sources, a man-made source and natural geological background. This indicates there is some relationship between the pollution levels, *EFs* and sources of these heavy metals.

### Potential ecological risk assessment (*RI*) of heavy metals in the sediment from Aibi Lake

To estimate the potential ecological risk posed by heavy metals in the sediments from Aibi Lake, we used methods by Hakanson (1980) [26]. Basically, we calculated the contribution to potential ecological harm of each single heavy metal and the potential *RI* of multiple heavy metals (Equation 3). The results suggest that the potential ecological risks of the seven tested heavy metals in the sediments of Aibi Lake were mainly caused by Cd, Hg and Pb. The *E*_*r*_^*i*^ contribution to the *RI*s of these heavy metals was 42.6%, 28.6%, and 24.0%, respectively, while the contribution of Zn, Cu, Ni and Cr to the *RI* was only 4.8%. Based on these calculations, the order of the single ratio of the tested heavy metals for the total potential ecological hazard is Cd>Hg>Pb>Zn>Cu>Ni>Cr.

This analysis also shows that in the sediment samples from Aibi Lake, the *E*_*r*_^*i*^ of Cu, Ni, Cr, and Zn were all below 40, placing these metals at low ecological risk level. Meanwhile, the average *E*_*r*_^*i*^ for Cd was 78, putting it at moderate pollution levels. Further calculations showed that about 45.7% of all the sediment samples were at a moderate ecological risk. For Hg, the average *E*_*r*_^*i*^ was 57, which places it at a moderate pollution level, and 36.4% of all the samples have a moderate potential ecological risk. Based on the comprehensive ecological hazard index of the heavy metals, we calculated the average *RI* values of all seven heavy metals, the analysis showed overall, 96.4% of the total samples were low ecological risk.

## Discussion

### Influence of human factors on Aibi Lake Basin

In this work, we found the *EF*, and the *I*_*geo*_ of the seven tested heavy metals in the sediment from the western, southern and southeastern parts of the lake were much higher than the northern, central and northeastern parts. This is most likely because the western part of the lake includes a river estuary and consists mostly of wetland, while the southern and southeastern parts of the lake were made up of farmland, townships and a traffic artery. Previous work has shown that human activity that causes pollution near the lakes, rivers and bays, such as industry, agriculture, urban living and road traffic, can significantly influence the chemical fraction of the water body and its sediment[50], former research have found that the Cd can be emission from agriculture by the fertilizer of potassica and phosphate, Pb and Hg from the burning of the soil from the traffics of the road, and Hg, Pb from the coal tar chemical and silicon chemical enterprises of the industries park around cities[15,51,52,53]. In this work, we found that in the western, southern and southeastern parts of Aibi Lake, the amounts of the heavy metals in the sediment were mainly influenced by the polluting emissions from industrial and agricultural activities, urban living and vehicular emissions. These heavy metals can then be transferred into Aibi Lake via river flow, rainfall and irrigation waste, and eventually accumulate in the lake sediment. There was further Pb, Hg, and Cd pollution from a nearby area. We also found where the *EF*, *I*_*geo*_, and *RI* of heavy metals were higher than other regions, which ended up being consistent with the identification of the sources through multivariate statistical analysis. This was also consistent with work by Darwin, in Canada [54], which has a high amount of tourism, shipping and industrial activities, Yangtze [55], and Dian Chi [56], in China, which have tourism, breeding and the social economy in the developed regions, that concludes that Pb, Cd and Hg in the sediment of the lakes studied were much higher than in areas with little human influence. Overall, this indicates that human activity has had a significant influence on the distribution of Pb, Cd, and Hg in the sediment in Aibi Lake.

The analysis showed the distribution of Cr, Ni and Cu in the sediments of Aibi Lake was mainly in the northern, central and northeastern parts. In these areas, the lake is distant from the township and traffic artery, and the main land types are desert and bare land, resulting in little human activity and indicating the quantity of these metals was mainly influenced by natural geological background. Former research also showed the weathering of the parent rock mineral and the soil parent material of the mountains can enrich the contents of the Cr, Ni, and Cu, then with the flow of the rain, these elements can be easily transferred into the water environment and eventually accumulated in the sediments of the lake [15,48]. Also, the levels of Cr, and Ni were significantly high and analysis suggests they were derived from the natural geological background. This concurs with the *EF*, and the *I*_*geo*_ values.

### Wind from Alataw Pass as a meteorological factor

**Fig 5:**
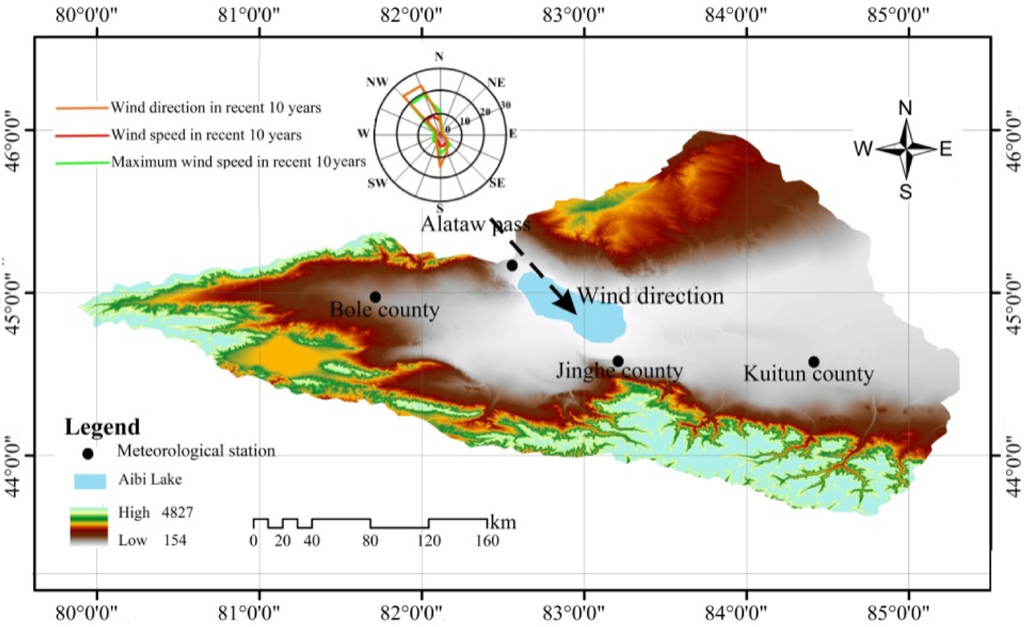
The annual wind direction of Aibi Lake Basin.

Alataw Pass is located in northwest China on the Kazakhstan border (Fig. 1) and is the widest and flattest part of northwest China. Due to the uniquely long, narrow and flat topography, it forms a famous windy pass. The maximum instantaneous wind speed goes up to 55 m.s^-1^ and 17 grades [25]. Former work by Mi et al. (2009) [57], found that the strong wind of the lake basin certainly contributes to the distribution of heavy metals in the water and sediments of the Bortala and Jing Rivers. We found a strong upwind direction for Aibi Lake, which may influence the heavy metals in both the water and the sediment of the lake. This analysis shows that the annual wind direction from the Alataw Pass in the past 50 years is heading northwest (Fig. 5), and the strong wind from Alataw Pass to the southeast may significantly influence the heavy metal accumulation in the water and, eventually, the sediment. Analysis of Aibi Lake shows the wind from the Alataw Pass significantly contributed to the distribution and accumulation of heavy metals in the sediment of the southern part of the lake, which is located in the downwind direction.

## Conclusion

In the research by analysis the distribution, sources and potential ecological risk of heavy metals in the sediment of a typical rump lake-Aibi Lake in an oasis in an arid area in northwest China, we can get the follow conclusions:

1. Descriptive statistics analysis determined that the average and maximum values were all higher than the background values of Xinjiang in northwest China. From the skewness, we ascertained the order to be Hg>Cd>Ni>Cr>Pb>Cu>Zn.
2. Multivariate statistical analysis shows that there were two main factors that influenced the amounts of the seven heavy metals in the sediment of the lake. PC1, consisting of Cd, Pb, Hg and Zn, was primarily influenced by man-sources, while PC2, consisting of Cu, Ni, and Cr, were mainly influenced by the natural geological background.
3. Calculation of the *EF* and *I*_*geo*_ placed Cd, Hg and Pb at low and partial pollution levels, while Zn, Cr, Ni and Cu were at no and low pollution levels; *RI* analysis showed that, when based on the *E*_*r*_^*i*^, the order of the seven heavy metals was Cd>Hg>Pb>Zn>Cu>Ni>Cr. Cd is the highest ecological risk factor, followed by Hg and Pb. Furthermore, the seven tested heavy metals are all low potential ecological risks.

This research showed recent economic development in the Aibi Lake Basin has negatively influenced the accumulation of heavy metals, this research also showed the strong winds from the Alataw Pass may contribute to the distribution and accumulation of the heavy metals in the sediment of Aibi Lake. This research can provide a scientific basis for heavy metal pollution, protection of the environment and the determination of reasonable policies concerning economic development in the Aibi Lake Basin and for the environmental protection of rump lakes in arid regions of northwest China.
